# Does demography have a role in measuring homelessness? Insights and approaches in the United States

**DOI:** 10.1553/p-5e53-9p3c

**Published:** 2025-12-17

**Authors:** Zack W. Almquist, Paul Hebert, Amy Hagopian

**Affiliations:** 1 Departments of Sociology and Statistics, University of Washington, Seattle, WA, USA; 2 Health Systems and Population Health, University of Washington, Seattle, WA, USA; 3 VA Health Services Research and Development, Seattle, WA, USA

**Keywords:** Homelessness, Demography, Measurement, Forecasting, Unhoused populations, United States

## Abstract

The global population experiencing homelessness has increased significantly over the last century. In 2021, the UN recognised homelessness as a violation of human rights, and urged member states to improve data collection and implement solutions for homelessness. This call presents both a challenge and an opportunity for demographers, especially in the US, to enhance their methodologies for counting and characterising this vulnerable population. Despite the escalating humanitarian crisis, the formal demographic literature engages little with the core demography of individuals experiencing homelessness, focusing instead on the social and behavioural aspects of the issue. A comprehensive review of this literature has identified only one article dedicated to measuring and enumerating people experiencing homelessness. Meanwhile, other disciplines are filling this gap, highlighting the need for demographic expertise on this issue. This article examines the definition and measurement of homelessness in the US, which has been estimated to affect over 770,000 individuals in 2024. It also discusses the demographic methods that can be used to study this population, and concludes with recommendations for the field.

## Introduction

The numbers and the demographic composition of individuals experiencing homelessness have expanded and diversified significantly over the past century. Due to multiple intersecting factors – including climate change, global pandemics, displacement due to war and conflict and a persistent shortage of affordable housing – homelessness has become a critical humanitarian issue of the 21st century. In 2023 alone, an estimated 63 million people worldwide were displaced by conflict and war, and another 7.7 million were displaced by disasters (House, 2007; Internal Displacement Monitoring Centre, 2024; Palinkas, 2020). Several recent incidents in the United States (US) have displaced thousands of people. For example, in 2019, 50,000 people in Northern California were displaced when the city of Paradise was consumed by a wildfire (Hennighausen and James, 2024). The ongoing housing crisis in the US (Colburn and Aldern, 2022) and the world (OECD, 2025a) further exacerbates these challenges. Recognising the severity of the issue, the United Nations (UN) General Assembly adopted resolution 76/133 in December 2021, formally acknowledging homelessness as a violation of human rights (United Nations, 2021). The UN calls on member states to gather better data on homelessness and implement programs to address the issue. While this call applies to all participating countries, it also offers an opportunity and a challenge to demographers worldwide, especially those in the United States, to improve their methods of enumerating and characterising one of the world’s most powerless, vulnerable and hidden populations.

Like in many other parts of the world, people experiencing homelessness in the United States are “the low end of a vast and growing wealth disparity” between social classes (Lee et al., 2010). Research on homelessness in the US has expanded significantly, particularly in social science and health fields. Much of this work examines structural causes, such as unemployment and the housing market, or individual causes, like mental illness and substance use (Main, 1998). However, recent studies have challenged the focus on individual factors, and have instead emphasised housing as the primary driver of homelessness in the US (Colburn and Aldern, 2022). Access to affordable housing is a growing issue across all OECD countries. Many low-income renters spend more than 40% of their income on rent, with the OECD average at 35.8%. However, this burden is significantly higher in the US and the UK, where low-income renters spend around 49% of their income on rent – the highest level among the OECD nations. In contrast, the rent burden in Canada sits just below the OECD average, at 35.4% (OECD, 2025a). To fully understand how both individual and systemic factors contribute to homelessness, it is essential to measure its scale, demographics and duration. However, despite the ongoing homelessness crisis in the US and globally, there is surprisingly little demographic research aimed at improving methods for accurately counting and analysing the unhoused population (OECD, 2025b).

In principle, demography is uniquely equipped to address this urgent call by the United Nations to action; currently, however, the field’s engagement on this issue is limited. Merli et al. (2023) splits the field of demography into two areas of study: (i) core demography, which focuses on measurement, enumeration, life tables, fore casting, modelling and other key demographic methodologies; and (ii) social and behavioural demography, which focuses more on issues such as migration, marriage, health and ageing. While there is interest in studying the social demography of people experiencing homelessness, including by examining the impact of poverty and inequality on this population, there is very little interest in studying the *core demography* of this population through the careful measurement, modelling or estimation of the number and composition (e.g., age or gender) of people experiencing homelessness. Demography has played only a minor role in the literature on people experiencing homelessness. Merli et al. (2023) provides a general framework for classifying the type of articles published in the field of demography that focuses on three major demographic outlets (*Demography*, *Population Studies* and *Population and Development Review*), spans the 1947–2022 period and covers 6252 articles. A general search for the word “homeless” or “poverty” on this corpus identifies 13 articles that deal with homelessness, but only three with the word “homeless” in the title. Among the 13 articles, only one would be classified by Merli et al. (2023) as undertaking core demography. This article by Lee (1989), titled “Stability and Change in an Urban Homeless Population”, focuses on the measurement of people experiencing homelessness. This article is the lone “core demography” article that works on the problem of the measurement and enumeration of people experiencing homelessness in urban areas in the United States. This paper is somewhat limited, as it only focused on cities, and it did not, as far as we can tell, have an impact on any of the core measures currently used by the federal government. This implies that in the field of demography, little effort has been made to improve methods for counting and measuring the unhoused population in the United States, and engagement with larger worldwide issues around homelessness has been even more limited. Meanwhile, a general search for “homeless” in Google Scholar produces 1,660,000 articles, suggesting that other fields are filling the gaps in methodology and in social behavioural demography.

In general, homelessness is not limited to the United States and is a global problem. The number of people experiencing homelessness worldwide is contested; however, a UN estimate claims that 150 million people are experiencing homelessness on any given night in countries that report statistics on the issue (Chamie, 2017). The enumeration of people experiencing homelessness is a complex and idiosyncratic process that differs not only among but also within countries, pointing to the need for large-scale reform and standardisation (OECD, 2025b). The field of demography is well-equipped to help provide and lead this reform. In this article, we will focus on the United States as an exemplar of the difficulties of capturing the size and composition of the homeless population even in well-resourced countries like the US.

**Figure 1:**
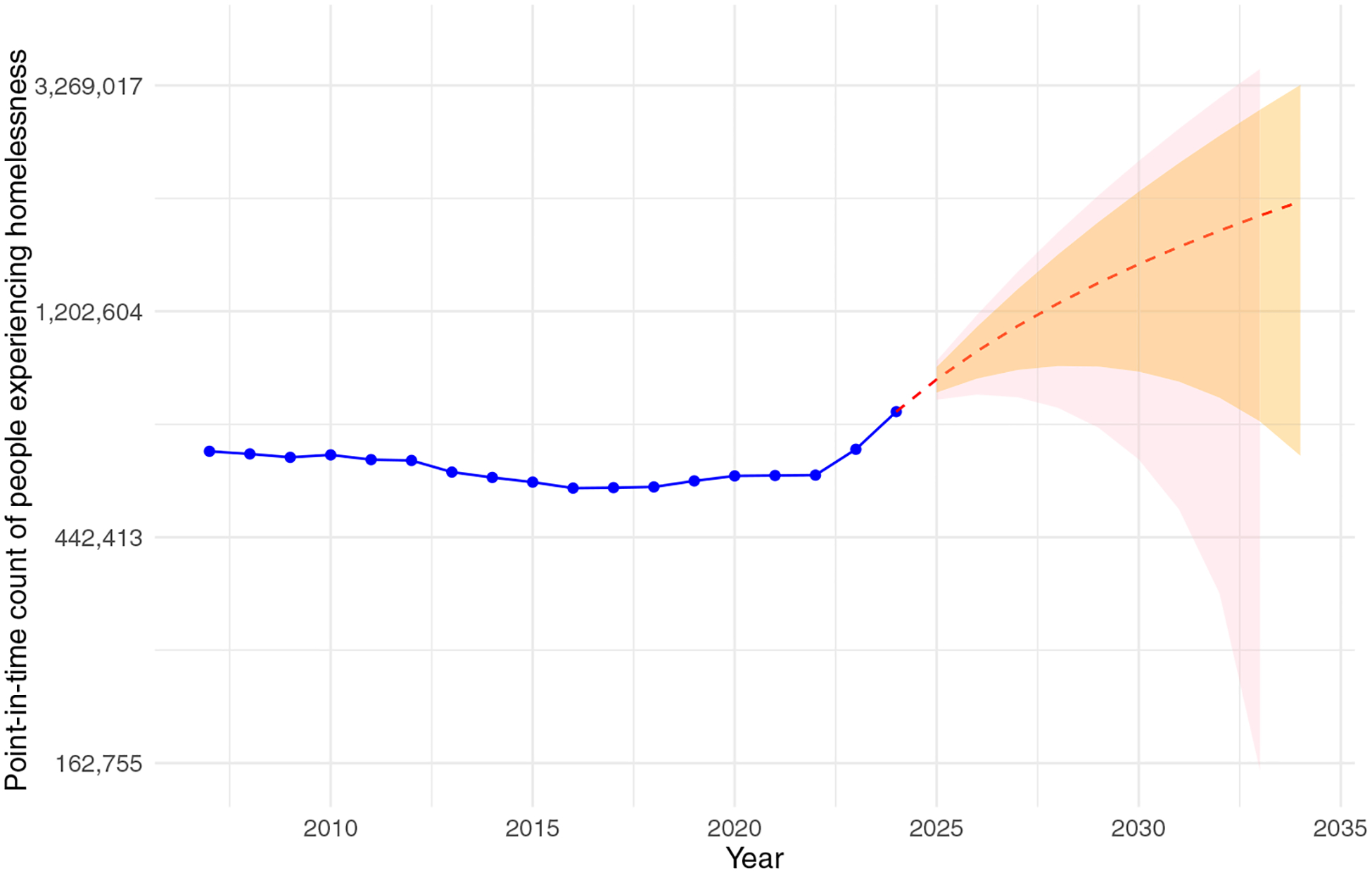
United States point-in-time count of people experiencing homelessness Source: Created by the authors based on the US Department of Housing and Urban Development’s national point-in-time data for people experiencing homelessness aggregated over all continuum of care geographic areas in the United States. The 10-year forecast is based on a simple exponential smoother. Notes: US point-in-time data for 2007 to 2024 with 2021 imputed using a simple ARIMA model (Shumway et al., 2017). The 10-year forecast is based on a simple exponential smoother. The red line is the middle estimate, the orange area is the 90% confidence interval and the pink line is the 80% confidence interval.

For example, the US reports that 1.4 million people experience homelessness, even if only briefly, over the course of a year (Olivet et al., 2021), and that 770,000 people experience homelessness on a given night (US Department of Housing and Urban Development, 2024). If we include the doubled-up population (e.g., couch surfing), one estimate would add another 3.7 million people to the homeless count in the US (Richard et al., 2024). Each of these counts is generally considered to be an undercount of the respective group (Lee et al., 2010). Even if we assume that all of these different counts are low, there are policy reports and academic articles estimating that the number of people experiencing homelessness on any given night or year ranges from 770,000 to 4.4 million. In Figure 1, we can see the US Department of Housing and Urban Development’s national roll-up of local jurisdictions from 2007 to 2024 (with 2021 imputed using a time series model), and a 10-year forecast showing that if current trends are maintained, more than a million people will be experiencing homelessness on any given night in the US. However, there is considerable uncertainty in these numbers, and the comparability of and the relationships between them are rarely made clear, as these metrics are collected and estimated using different data sources and methods. This points to an extensive set of problems the demographer’s toolkit is well-suited to address.

In this article, we examine the demography of homelessness in the US as a case study, illustrating how demographers can approach this issue domestically and, potentially, globally. This article is organised as follows: (1) we review the definition of homelessness in the US; (2) we provide a history and discussion of the methods used in the US to measure homelessness (including the doubled-up population); (3) we review the classic demographic measures applied to the unhoused population (i.e., length of time in and out homelessness and mortality); (4) we provide a discussion of our findings; and (5) we conclude with a summary and future directions.

## Definitions of homelessness in the US

This article focuses on the US federal definition of homelessness provided by the US Department of Housing and Urban Development, the agency responsible for administering federal housing and urban development laws. It is well established that the definition of homelessness drives reports on the size and composition of the unhoused population (Sullivan, 2023). Still, to allow for a practical and effective discussion of the demography of this population, we follow governmental definitions. Other agencies and countries follow other definitions of homelessness, such as the European Typology on Homelessness and the Housing Exclusion framework (Amore et al., 2011).

In the US, the McKinney-Vento Homeless Assistance Act broadly defines homelessness (for the purposes of the federal government and many local jurisdictions) as including individuals and families lacking a fixed, regular and adequate nighttime residence; those living in shelters, transitional housing or places not meant for habitation; individuals at imminent risk of losing their housing within 14 days with no subsequent residence having been identified; and unaccompanied youth and families with children who are unstably housed or facing persistent housing instability. See Table 1 for a detailed breakdown and Housing and Urban Development (2023) for further formal details.

Notice that the definition of homelessness in Housing and Urban Development (2023) does not include people who are couch surfing, staying with a friend or family or even paying for a hotel room. This definition of homelessness contrasts with that used in much of Europe and Canada, which considers this population part of the unhoused community under their Provisionally Accommodated category (Gaetz et al., 2012). We will engage more with this “doubled-up” population in the next section.

## Measuring homelessness in the US

The measurement of homelessness in the US (and worldwide) is a multifaceted process that has been undertaken by academics, federal and local agencies, nonprofits and advocacy groups for various purposes, including to inform strategic action. In this section, we first lay out the history of people experiencing homelessness in the US and then review administrative data on the unhoused population. Next, we discuss some of the work on the doubled-up population, who are not considered homeless by the federal Housing and Urban Development agency, but are typically considered homeless by public health officials and local agencies. Finally, we consider the most challenging group to count, i.e., the “unsheltered” or those living “rough” in tents, on park benches, etc. In this last section, we discuss the approaches that have been applied or considered, and some of the pitfalls and potential advantages of each method.

### History of counting the unhoused population in the US

The US has a long history of counting people experiencing homelessness. Government agencies, academic researchers and homeless care advocates have done this work. Below, we discuss and compare these efforts over the last century, with a final comment on the role of statistical sampling versus censuses of people experiencing homelessness in the US context.

#### History of governmental counting of homelessness

The counting of people experiencing homelessness in the United States began in the 1970s and 1980s. In the 1980s, homelessness in the US came under the national spotlight when advocates – specifically, the National Coalition for the Homeless – described it as an epidemic afflicting millions of people (e.g., Hombs and Snyder, 1986). Homelessness became a contentious political problem, motivating a debate on how to measure the homeless population in the US. In response, the Housing and Urban Development agency sought to obtain high-quality counts of the homeless population.

In 1983 and 1984, the Housing and Urban Development agency conducted a series of surveys (Bobo, 1984; Burt and Cohen, 1989), which were later followed by two major studies: (1) the US Department of Agriculture^1^ 1987 study that aimed to enumerate a national sample of unsheltered people in the US; and (2) a study that presented the prototype method for systematic local-enumerations based on stratified samples of microgeographies, piloted in Chicago. The USDA study attempted to count the number of people experiencing homelessness and record their demographics in 20 major cities across the US. It is considered the first nationally representative dataset for homelessness in the US (Poulin et al., 2008). Chicago’s systematic local enumeration method was developed and published by Rossi et al. (1987), and has influenced modern methods for estimating the size and composition of homeless populations.

The 1987 McKinney Act tasked the US Census Bureau with counting the homeless population during its regularly scheduled census; the Act assigned the largest share of the responsibility for guiding this piece of the US census count to the Housing and Urban Development agency (Lee-Anderson, 2019). The US Census Bureau then followed up the Chicago study during the 1990 census, expanding the count to five major US cities, which are collectively known as the S-Night sample (where “S” stands for both street and shelter; Barrett et al., 1990). This experiment, run by the US Census Bureau, is the basis for the point-in-time count currently used by the Housing and Urban Development agency (despite the critique that the methods applied in the different sites were so inconsistent that the results could not be compared). The five cities, Chicago, Los Angeles, New Orleans, New York and Phoenix, were chosen to represent various regions and weather conditions, and include the two cities believed to have the largest homeless populations (New York and Los Angeles).

In 1999, Congress directed the US Department of Housing and Urban Development to create a standardised approach for reporting national homeless counts. This process led to the establishment in 2005 of the continuum of care area units or communities and point-in-time counts, which have been used since 2007 (US Department of Housing and Urban Development Office of Community Planning and Development, 2009). Thus, the US Department of Housing and Urban Development’s *Annual Homelessness Assessment Report* has become the authoritative estimate of the size of the nation’s homeless population. The Housing and Urban Development agency’s methodology for counting the unhoused population includes estimates on a given night (i.e., the point-in-time count) and estimates over a year (i.e., the “annual prevalence”) (Metraux et al., 2016).

#### Critiques of governmental methods for the counting of people experiencing homelessness

The primary critique of the traditional point-in-time method pertains to its propensity to undercount homeless populations. This happens because individuals must be visible to be counted, but environmental conditions often impede visibility, and many individuals prefer to remain hidden (and succeed in doing so). Moreover, the Housing and Urban Development agency restricts the definition of homelessness, and the enumeration methods used by different jurisdictions tend to vary. Critics also note the changes over time in the inclusion criteria, such as in the classification of transitional and permanent housing services, which can make these statistics difficult to compare and interpret, or even opaque (Hopper et al., 2008; Institute of Medicine, 1988; Lee-Anderson, 2019; National Law Center on Homelessness and Poverty, 2017; Schneider et al., 2016). The subjectivity, bias and precarity of pointin-time estimates have been shown to significantly affect the counts (Golinelli et al., 2015). For instance, New York’s Department of Homeless Services interviews visible homeless people between midnight and 4 AM on a given night each year, and has conducted experiments to estimate the number of people missing from the count. The results showed that an estimated 30%–40% of unsheltered people experiencing homelessness were in a location that point-in-time surveyors likely missed (Hopper et al., 2008).

Advocates have increasingly pointed out discrepancies between the point-intime estimates and reality. They have argued that conventional estimates minimise the problem, leading to misinformation and insufficient policy responses. There is considerable evidence supporting their claims: for instance, a general population sampling approach conducted in Los Angeles found that adding the unsheltered homeless persons who were missed in a point-in-time enumeration increased the estimated size of the total population by more than 20% (Agans et al., 2014).

#### Academic research studies estimating the number of people experiencing homelessness

Several one-off small area estimation case studies of homeless populations have been conducted in major US cities (e.g., Los Angeles; Berk et al. (2008)), and intensive surveys of people experiencing homelessness have been undertaken (e.g., US Census S-Night count). Berk et al. (2008) used the 2004 pre-continuum of care mandated point-in-time count to build estimates of county-, city- and census tract-level homelessness for Los Angeles County. The researchers sampled data for census tracts at two waves and applied a random forest approach to modelling and aggregating tract sample data (totals are estimated using a Horvitz-Thompson estimator). Other researchers have used the US Census S-Night sample to make small area estimates (e.g., Bentley, 1995; Lee and Price-Spratlen, 2004; Wright and Devine, 1992).

A handful of longitudinal studies exist, such as Link et al.’s (1995) study of the five-year prevalence rates of homelessness. Given the pervasiveness of the problem, surprisingly limited use has been made of the nationwide homeless count data (available from the Housing and Urban Development agency for academic research). This is partly because the data are not linked to major governmental datasets like the US census. Notable exceptions include Byrne et al. (2013) and subsequent follow-up research on community factor predictors of homelessness (Byrne et al., 2013). However, the only significant collection of data on homelessness counts is the US Department of Housing and Urban Development’s decentralised and multimethod “continuum of care” census count. More recently, Agans et al. (2014) suggested using a phone-based survey approach to improve the standard point-intime count, and Bergmann et al. (2021), in a policy report to Hennepin County, Minnesota, proposed using a spatial sampling approach for future point-in-time counts.

#### Research studies by advocacy and non-profit service organisations attempting to estimate the number of people experiencing homelessness

These estimates include posthumous assessments, recorded uptake of shelter and homelessness services, location sampling and innovative approaches like “plantcapture strategies”, among others (Golinelli et al., 2015; Hopper et al., 2008). Critics of the point-in-time count point to its origins as a local non-profit-led community effort to bring awareness to the problem of people experiencing homelessness, rather than as a rigorous and complete count of the unsheltered population.

King County^2^ was among the first jurisdictions to organise a local point-in-time count, which was conducted by the Seattle King County Coalition on Homelessness as a community engagement and advocacy exercise (Seattle/King County Coalition on Homelessness, 2024).

#### Discussion of relying on census counts versus sampling for policy decisions

The 1999 Supreme Court case of “Department of Commerce v. United States House of Representatives” addressed the use of statistical sampling in the US census. The case questioned whether the Census Bureau could employ sampling instead of a complete headcount to estimate population. In a 5–4 decision, the Court ruled against statistical sampling for apportionment, stating that it violated the constitutional requirement of an “actual enumeration”. This decision set a precedent, affirming that a census for apportionment must involve a direct headcount, regardless of its accuracy (and, therefore, the 2000 census was limited to a headcount, even though demographers cautioned it would undercount minority populations; Anderson and Fienberg, 2002). Despite its methodological flaws, this historic ruling has influenced agencies to privilege a direct headcount of homeless populations in outdoor locations (e.g., woods, streets and parks), emergency and transitional housing, soup kitchens and regularly scheduled mobile food vans (US Census Bureau, 2023). Critics (Meyer et al., 2023) have demonstrated that US census microdata included a number of duplicate entries. Furthermore, Meyer et al. (2023) showed that around 90% of individuals in shelters appear in the US census. However, the unsheltered point-in-time count is conducted much more like the American Community Survey or the Current Population Survey, serving to supplement service user Homeless Management Information System data rather than the actual US decennial census. This is similar to the American Community Survey, which provides yearly demographic estimates for the US rather than 10-year estimates for drawing political boundaries.

### Databases and administrative data: Measuring the population who use services in the US

In the US, administrative data capture the number of individuals who are “lucky” enough to access emergency shelters and other temporary housing structures (e.g., tiny homes). This section discusses how these individuals are measured in the US context. First, we describe the US Department of Housing and Urban Development’s administrative structures and databases resulting from the 1994 consolidation of homeless care jurisdictions. We then review the Housing and Urban Development agency’s database standards enforced through the Homeless Management Information System.

#### Continuum of care

In 1994, the US Department of Housing and Urban Development began requiring each jurisdiction (city, county or other similar entity) to bring together service providers to submit a comprehensive “continuum of care” funding application, replacing its practice of contracting directly with individual community providers. These local coalitions provide stable administrative units for US homeless services and incentives to coordinate homelessness planning in specified areas. Since 2007, the US Department of Housing and Urban Development has been requiring and guiding local continuum of care agencies to make estimates of the homeless population. Each continuum of care adapts the Housing and Urban Development agency’s standard approach to counting those using services (typically emergency shelters) and those living unsheltered.^3^

**Figure 2:**
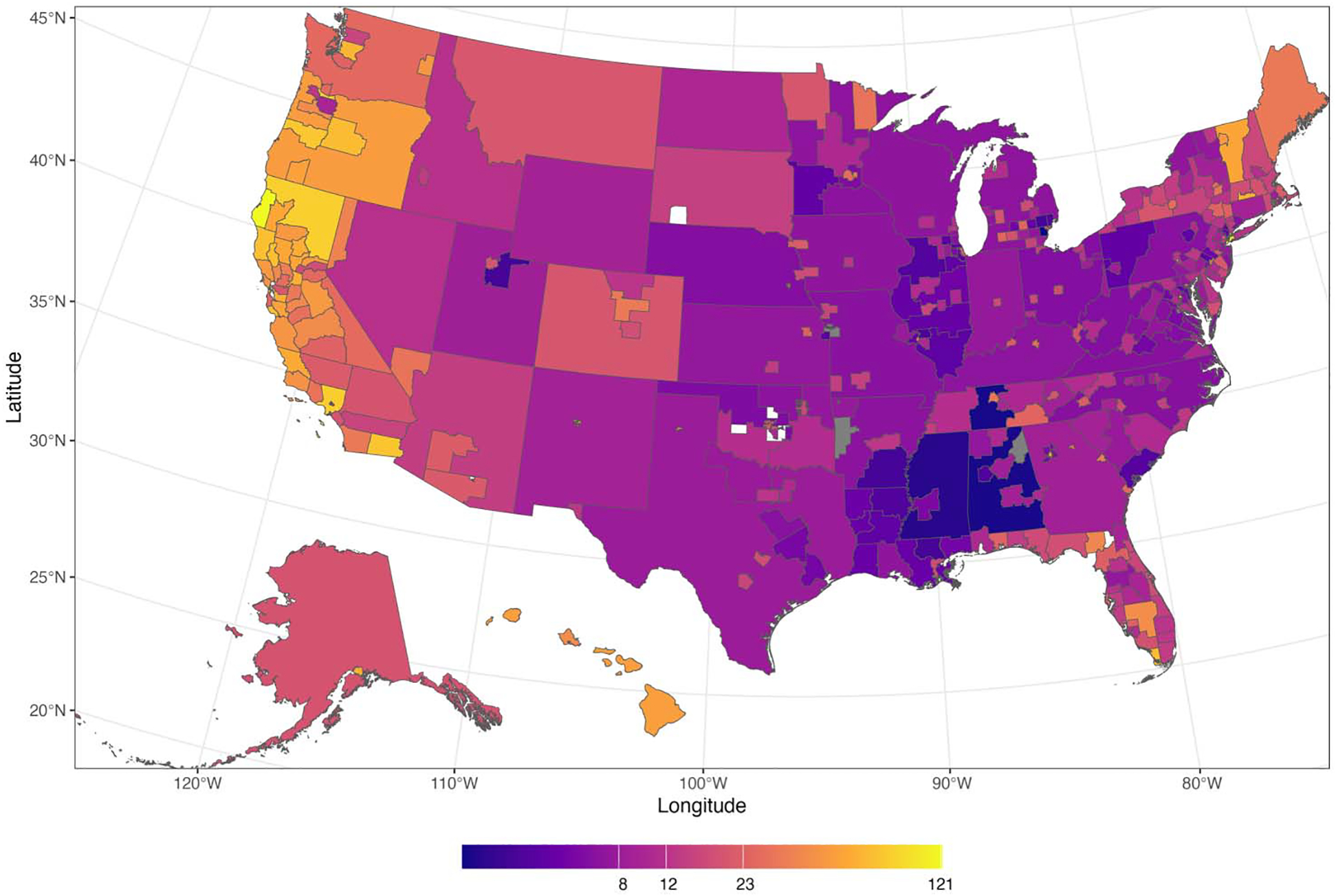
Choropleth map of people experiencing homelessness in the US Source: Created by the authors based on data from HUD PIT & HIC Inventory Count (2023) and the US Census Bureau (2022). Notes: The US continuum of care population of people experiencing homelessness per 10,000 people. US Department of Housing and Urban Development’s homelessness care jurisdictions (continuum of care) for the United States in 2023. Total people experiencing homelessness choropleth map. Note that the states with the lowest incomes do not have the highest homelessness rates; this is consistent with the work of Colburn and Aldern (2022), who concluded that homelessness is largely a reflection of the availability of affordable housing rather than a household income problem.

In 2023, 381 federally designated jurisdictions were responsible for caring for and counting the unhoused population (Housing and Urban Development, 2023). The combined point-in-time count was tallied in the 2023 US Department of Housing and Urban Development report to Congress, which concluded that a total of 653,104 people were experiencing homelessness at a given point in time, up 12% from 2022, with 40% of these individuals experiencing raw, unsheltered homelessness (sleeping “rough”) in places not meant for human habitation (HUD PIT & HIC Inventory Count, 2023), and the remaining 60% sleeping in overnight shelters. The Housing and Urban Development agency’s definition of homelessness is shown in Table 1. The 2023 count of people experiencing homelessness is illustrated spatially in Figure 2. Almquist et al. (2020) demonstrated and discussed a method for linking continuum of care areas to US census administrative units for social science and policy analysis, given that these jurisdictions do not align by default with US census geographies (e.g., blocks or tracts).

#### Homeless Management Information System

The Housing and Urban Development agency requires continuum of care areas to follow the data collection and administrative control standards of its Homeless Management Information System database (Culhane, 2004). The Homeless Management Information System provides a count of unhoused people registered through service providers, generating data for the federal reporting system. Specifically, the US Department of Housing and Urban Development offers a yearly series of data standards, updated through the Homeless Management Information System Data Standards Manual. The Homeless Management Information System Data Standards Manual assists Homeless Management Information System leads/system administrators, continuum of care administrators and Homeless Management Information System end users in their data collection and reporting efforts. It mandates the essential Homeless Management Information System data elements to ensure compliance with the Housing and Urban Development agency’s and the federal partners’ participation and reporting mandates (Housing and Urban Development, 2022). Every continuum of care maintains a Homeless Management Information System database, which conforms to this set of standards.

### Doubled-up population in the US

In much of the world – and among public health advocates in the US – people who live together for reasons of economic hardship (i.e., the “doubled-up” population) would be considered homeless. The term “doubled-up” refers to a living arrangement in which individuals or families are staying temporarily with friends, relatives or others because they cannot afford their own housing or have lost access to stable housing. This situation is created by financial hardship, eviction, job loss, family disintegration, legal trouble, medical problems, domestic violence or other crises.

**Figure 3:**
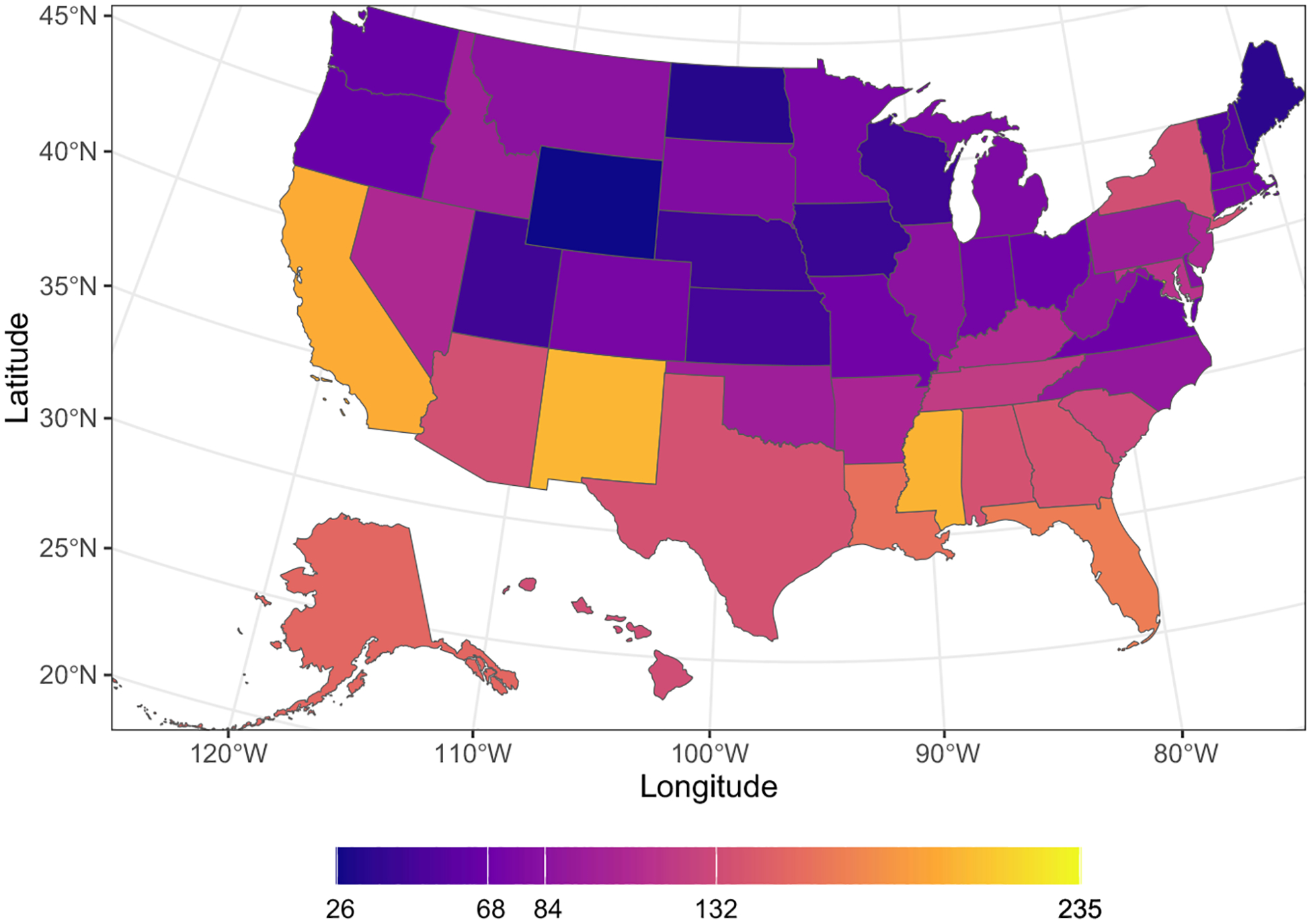
Doubled-up population by state in 2019 Source: Created by the authors based on data from Richard et al. (2024). Notes: Doubled-up population estimated by Richard et al. (2024) using US Census American Community Survey data for 2019 by state.

In the US, the McKinney-Vento Act definition of homelessness does not include those who double-up (Richard et al., 2024). While this is another area demographers have not engaged in, there have been recent developments in the housing literature, including employing the US Census American Community Survey micro-use data combined with the IPUMS relationship file (Ruggles et al., 2024) to quantify the doubled-up population in the US (Richard et al., 2024). Richard et al. (2024) provided their 2019 estimates by micro area and state (see Figure 3 for a state-level illustration of the potential doubled-up population in the US).

**Figure 4:**
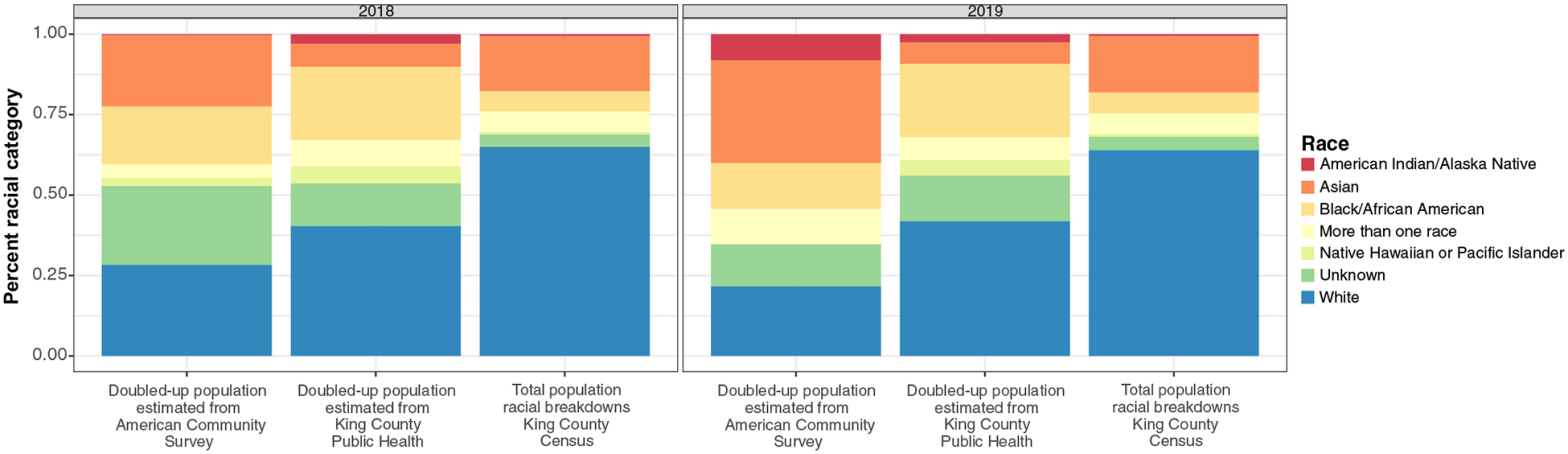
Estimates of the doubled-up population in King County, Washington, in 2018 and 2019 Source: Created by the authors based on data from the American Community Survey (Ruggles et al., 2024) and King County Public Health. Doubled-up racial and ethnic comparisons for King County, Washington, in 2018 and 2019 were estimated from the King County Public Health database with deduplication and the Richard et al. (2024) American Community Survey method. Notes: Doubled-up population by race for 2018 and 2019 as estimated from the King County Public Health database and the American Community Survey compared to the total race breakdowns computed by US Census data.

Research on the doubled-up population is highly fraught due to competing definitions and limited data. National data suggest that in 2015, more than 1.6 million public school students were experiencing homelessness, and nearly a million were doubled-up (Kena et al., 2016). For an example of how local estimates may differ from US census-derived estimates or public school estimates^4^, see Table 2, which compares the King County Public Health database count of doubled-up individuals in King County, Washington, as reported to health services in the area to the American Community Survey method in Richard et al. (2024), which reports orders of magnitude more doubled-up people. Furthermore, Figure 4 illustrates the differences in racial composition depending on whether the data source for the doubled-up population is the American Community Survey method or the King County Public Health record.

### Current and alternative methods for estimating “street” homelessness in the US

There is general agreement that most estimates of the unsheltered homeless population in the US are undercounts, and that better methods would improve our understanding of those not using a shelter on the given night of measurement. While there is no universally accepted method for counting the number of people living outside (see Figure 2 for an example), standard practices are used across most continuums of care. This approach has become known as the “unsheltered point-in-time count” or the visual census of people experiencing homelessness. The visual census is typically performed between midnight and 6 AM on one night in the last two weeks of January (as mandated by the Housing and Urban Development agency). The lead organisation in the continuum of care brings together volunteers and maps (now often geo-coded apps). The volunteers then go out in the dark with clipboards (or pads) and record every person they see who looks like they are sleeping outside (or in a car, or on the bus) in that time period. This is followed up by a two- to four-week demographic and needs-based survey. This survey is sometimes based on a spatially stratified convenience sample, or is just taken from provider records. See the Housing and Urban Development agency’s methods manual (Housing and Urban Development, 2023) for a list of all the methods that can be used.

Here, we present five major categories of alternatives to the traditional US Department of Housing and Urban Development-endorsed visual census for arriving at an unsheltered point-in-time count: (1) mining administrative databases (e.g., the Homeless Management Information System), accompanied by passive data collection; engaging the service community to directly report on people experiencing homelessness; (2) combining survey and routine administrative data; (3) improving the visual unsheltered point-in-time count through technology; (4) conducting online surveys; and (5) conducting surveys over social networks (peer referral). We discuss each method below, and provide a table summarising each of the approaches in Table 3.^5^

#### Databases, passive data collection and engaging the service community

Tsai and Alarcon (2022) proposed two leading alternatives to the visual census point-in-time count. (1) The first approach, Continuous Monitoring and Technology Integration, would implement a nearly real-time monitoring system using technology, such as mobile apps and data analytics, with the aim of providing a continuous, comprehensive enumeration of people experiencing homelessness. This real-time data analytic strategy would help to overcome the limitations of a one-day count (which takes place in January, when people are least likely to be out on the street, even if they are homeless) and capture fluctuations over time. However, passive monitoring (e.g., review of cell phone records) has significant limitations. One is methodological, as many homeless people do *not* have cell phones, or they lose them, share them or turn them off to preserve battery. Moreover, disambiguation between the unhoused population, service workers and the housed population, who are often all in the same location, can be an almost intractable problem. Another major issue with this process is the matter of consent, as people generally object to being monitored without their knowledge. (The authors conducted a series of focus groups with people experiencing homelessness in King County in 2022 to ask about this issue, and it was abundantly clear that unhoused people do not like the idea of monitoring cell phone signals to obtain counts of people experiencing homelessness. People in our focus groups specifically called it “Big Brother-esque”^6^ monitoring.) (2) The second approach, Collaboration and Community Engagement, proposes involving local communities, social service organisations and volunteers in data collection with the aim of enhancing the count’s accuracy while building awareness and political and material support. This process is widespread in street counts, as an organisation needs many people to cover a given continuum of care (historically, King County deployed 1000 volunteers as street counters during a single evening). This approach may help to identify and reach homeless individuals not easily accessible during traditional counts. Although it has required significant and expensive organisational collaboration, the community engagement that has been built through this lively annual census enumeration event has served a purpose.

Ward et al. (2022) introduced a method of geographic subsampling in the county of Los Angeles, California. While this strategy does not replace the point-in-time count, it provides a framework for gathering demographic data using a spatial skip sampling procedure (randomly sampling spatial areas and then entering each space to interview every other or every fifth person experiencing homelessness that the interviewer comes across). By using individual identifying information, this method provides estimates of the demographic proportions of people experiencing unsheltered homelessness, and may be expected to generate very high-quality proportional estimates. One important limitation of this approach is that the interviewer walks up to the person directly, putting pressure on the respondent to participate in the survey, which raises critical consent issues. Moreover, this strategy is resource intensive, given that substantial staffing would be needed to implement it over a large geographic area like Los Angeles, California, or even King County, Washington.

Meyer et al. (2023) attempted to evaluate how well the current census and American Community Survey cover the population of people experiencing homelessness in the US. They demonstrated that most people sheltered in emergency settings are counted in the Homeless Management Information System data (around 90%). It is unclear whether this method can be employed for non-archival data analysis. Other national surveys could also be tried, but we are unaware of any efforts to do so in the US.^7^

#### Survey and administrative data

The plant-capture/recapture method is an innovative approach borrowed from ecological research and population biology (Hopper et al., 2008). In the context of estimating unsheltered homelessness, this method involves placing discrete markers (embedded human decoys) in locations where homeless individuals are known to frequent, and recapturing these markers at a later date. To do this, staff members who have received training are tasked with assuming the role of decoys. They are directed to dress and behave as if they were homeless. Afterwards, they provide feedback to the continuum of care regarding whether they were included in the count on the designated night. An estimate of the unsheltered homeless population can be calculated by determining the rate of marker recapture and using statistical models. This method can improve the estimate of the total unsheltered homeless population, but it does not provide demographic breakdowns or a proper statistical estimator.

The capture-recapture methods (sometimes called multiple-list methods, see, for example, Weare, 2019) are statistical approaches derived from ecological and wildlife population studies (Williams and Cheal, 2002). Using capture-recapture methods to estimate people experiencing unsheltered homelessness involves two or more separate counts of the same population within a defined time frame, allowing for an estimation of the total population size through mathematical modelling. Capture-recapture can address the issue of point-in-time undercounting by providing a statistical correction. Accurate capture-recapture methods require data from multiple sources, such as outreach teams, service providers and independent counts, which can be logistically challenging to coordinate. Currently, the method does not estimate demographic characteristics, and it typically involves conducting a separate survey rather than just using administrative logs.

Demographic post-point-in-time surveys sample individuals counted after the traditional point-in-time count. They are typically run as a spatial sample (e.g., Los Angeles County) or a convenience sample at day centres, food banks, etc. These surveys gather detailed demographic and situational information, shedding light on the characteristics and needs of the unsheltered homeless population. Surveys provide valuable individual-level data, allowing for a deeper understanding of the homeless population’s characteristics. These surveys are fielded to obtain general information on the demographics and needs of people living outdoors.

#### Visual unsheltered point-in-time technology improvements

Geographical Information System tools offer a robust set of resources to improve the accuracy and value of the unsheltered point-in-time count. Incorporating spatial analysis, geocoding, mobile data collection and integrated data recording into classic visual unsheltered point-in-time counts can improve the overall counting process. Adding apps and automated geocoding has improved the accuracy and reliability of classic unsheltered point-in-time count locations.

#### Volunteer geographic information

Chien et al. (2024) and others have started to look into volunteer geographic information data, such as 311 reports by local citizens in the City of Los Angeles or outreach records/activities by service providers correlated with the visual pointin-time census. Chien et al. (2024) demonstrated that the volunteer geographic information data for Los Angeles predict unsheltered homelessness at high levels. Suttona et al. (2024) has also examined how outreach records/activities by service providers can be used to understand the spatial distribution of people experiencing unsheltered homelessness.

#### Online surveys and network methods

Network scale-up methods (Laga et al., 2021) have been used in the academic and public health literature to enumerate people experiencing homelessness (Killworth et al., 1998). This method takes a random sample from a known population (e.g., landline, cell phone or address-based sample) and asks respondents how many people they know in each category, including the priority population of people experiencing unsheltered homelessness. Recent improvements in this method could be employed to better understand the unsheltered population (see, for example, the work of Feehan and Salganik (2016)). In addition, scale-up methods have been employed with online surveys to estimate offline populations using a random sample of Facebook users (Feehan and Cobb, 2019). This strategy has the potential for estimating the unsheltered population in the US. Maas et al. (2020) and Giraudy et al. (2021) demonstrated how Facebook data could be used to sample displaced people, employing a post-disaster case in Australia to produce estimates comparable to those of the UN for the total number of people displaced during a bushfire. This method could also be adapted to estimate the total number of people experiencing unsheltered homelessness.

#### Snowball, respondent-driven sampling and other network approaches

Two sampling designs routinely used in social science and public health research are *snowball sampling* and *respondent-driven sampling* (RDS). Snowball sampling (where respondents supply names of their known contacts) is typically used for either “obtaining a nonprobability sample through an unspecified network search process or constructing a frame from which to sample” (Handcock and Gile, 2011). In either case, it is not formally the same process as respondent-driven sampling, which is, as Handcock and Gile (2011) described, a link-based sampling approach. In other words, respondent-driven sampling is a process for formally sampling (i.e., randomising) the social network rather than relying on a pure convenience sample of the target population. In general, respondent-driven sampling is a method for both data collection and statistical inference that relies on selecting a small number of initial participants (“seeds”) from the target population who are then asked to recruit their contacts in the target population (this is usually followed with incentives), and that includes a randomisation element. This procedure is repeated until a desired sample size is reached. The typical recruitment size is three per a given contact’s personal network. Because this process is known to over-sample people with many connections, the sample is reweighted to provide an unbiased (or minimally biased) estimate. This method has been employed as an official method for measuring the unsheltered homeless population in the continuum of care of King County, Washington, in 2022 and 2024 (Almquist et al., 2024b).

## Classic demographic measures applied to the unhoused population

Beyond measures of the size and composition of the unhoused community, policymakers, public health researchers, demographers and other social scientists continue to be interested in applying classic demographic measures of birth (entry into homelessness) and death (exit from homelessness or actual death) to the process of experiencing homelessness. Below, I discuss the major themes considered in the literature regarding time spent homeless and general mortality.

### Duration and life course of homelessness

**Figure 5:**
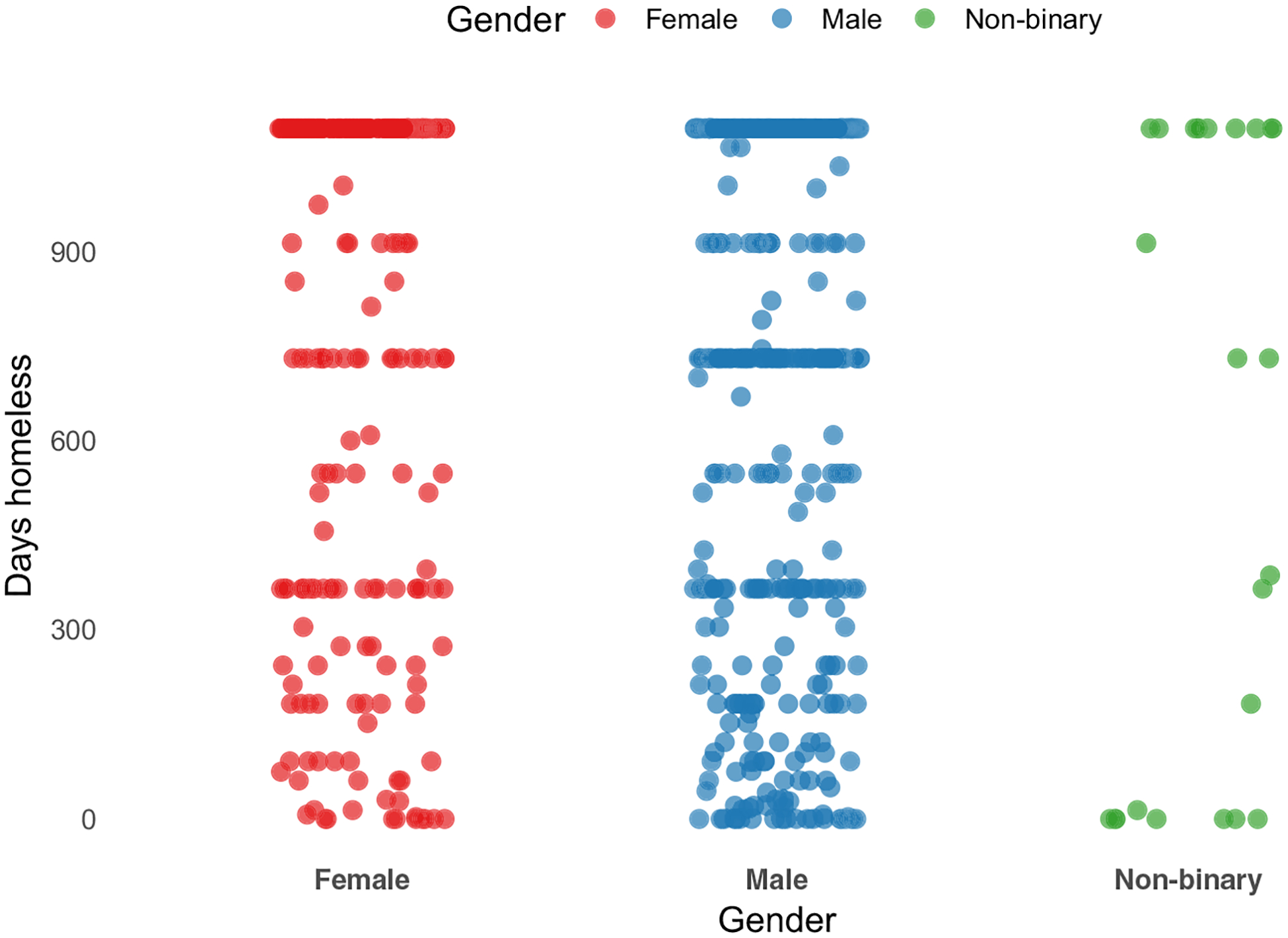
Distribution of days homeless by gender, King County, Washington Source: Created by the authors based on self-reported days spent homeless in the last three years by gender in a population-representative sample of King County, Washington, collected in 2023 (Almquist et al., 2024b). Notes: Grid plot of days spent homeless in the last three years by gender in King County, Washington, in 2023.

Homelessness is a dynamic experience shaped by events across the life course, including early life disadvantages, socio-economic disruptions and structural inequities. Life course theory suggests that exposure to risks and protective factors during key developmental stages shape health and social outcomes later in life (Elder Jr et al., 2003). For example, from Figure 5, we can see that there is considerable heterogeneity by self-reported gender in the number of days spent homeless in King County, Washington, in 2023. There are limited data on the life courses of people experiencing homelessness. Still, new data are being collected by a continuum of care and by a number of researchers that could improve our understanding of the demographics and experiences of those living unhoused.

Life course research highlights how certain groups, such as birth cohorts or those facing systemic barriers, are at heightened risk of experiencing homelessness (Willekens, 1999). Spells of homelessness vary widely, with some individuals facing brief episodes while others endure chronic homelessness, often cycling in and out of unstable living conditions (Kuhn and Culhane, 1998). The duration of time spent homeless has profound implications, as longer spells are associated with worsened health outcomes, deeper poverty and increased barriers to recovery (Culhane and Kuhn, 1998). Understanding the trajectories of homelessness over the life course is crucial for designing interventions that address immediate needs and root causes.

Recently, older adult homelessness in the US has been linked to a birth cohort effect, with individuals born between 1955 and 1965 being disproportionately represented since the 1980s. This trend, now extending to 2020, highlights the growing issue of over-65 homelessness, which poses significant challenges for healthcare and social services (Byrne et al., 2024). Furthermore, the critical work of Kuhn and Culhane (1998) and Culhane and Kuhn (1998) used event history analysis to assess the length of stay and rate of readmission to emergency shelters among homeless adults from New York City (1987–1994) and Philadelphia (1991–1994). Discrete-time logistic hazard regression analyses revealed that, in general, being older, being Black, having a substance abuse or mental health problem or having a physical disability significantly reduced the likelihood of exiting the shelter. In both cities, people entering shelters in their later years stayed longer, although some individuals had shorter episodes following subsequent admissions. Generally speaking, the authors found that for most people, homelessness was a one-time phenomenon, at least over a three-year follow-up period, and typically that lasted less than a month. Much of this work has been replicated on veterans; see, for example, Tsai and Byrne (2019), who looked at the veteran homeless program among a “first-time” entry cohort of veterans over two years, and found that most veterans made relatively brief use of Veterans Affairs homeless programs over the study time frame. Furthermore, Byrne et al. (2015) found that among veterans who initially screened positive for homelessness and then completed a follow-up screen 6–12 months later, roughly 85% did not report any housing instability at the time of the follow-up screen.

### Mortality in people experiencing homelessness

In the public health literature, the causes and health consequences of homelessness, especially mortality, are striking (Omerov et al., 2020). Recently, Fowle and Routhier (2024) demonstrated there was an increase in the mortality rate of people experiencing homelessness from 2011 to 2020. An active project is underway to collect death counts of people experiencing homelessness during the COVID-19 pandemic by county for the US (Fowle and Gray, 2024). The results have revealed stark disparities in the life courses of housed and unhoused people. Still, in this report, the authors were only able to obtain death counts for people experiencing homelessness in 79 counties for 2018 (out of 3144 total counties in the US).

While there is no systematic review of the role of homelessness in the age of death, there is a consensus that it dramatically shortens life expectancy. For example, the adult homeless population crude mortality rate for Boston, Massachusetts, was 1439.5/100,000 person-years, which translated to a mean age of death of 51 years old (Funk et al., 2022). Nicholas et al. (2021) computed crude death rates and age- and gender-adjusted mortality rate ratios for people experiencing homelessness in Los Angeles County. A cohort study conducted in Philadelphia, Pennsylvania, revealed that between 1985 and 1988, the age-adjusted years of potential life lost before age 75 were 3.6 times higher among homeless individuals than among the general population (Hibbs et al., 1994).

**Figure 6:**
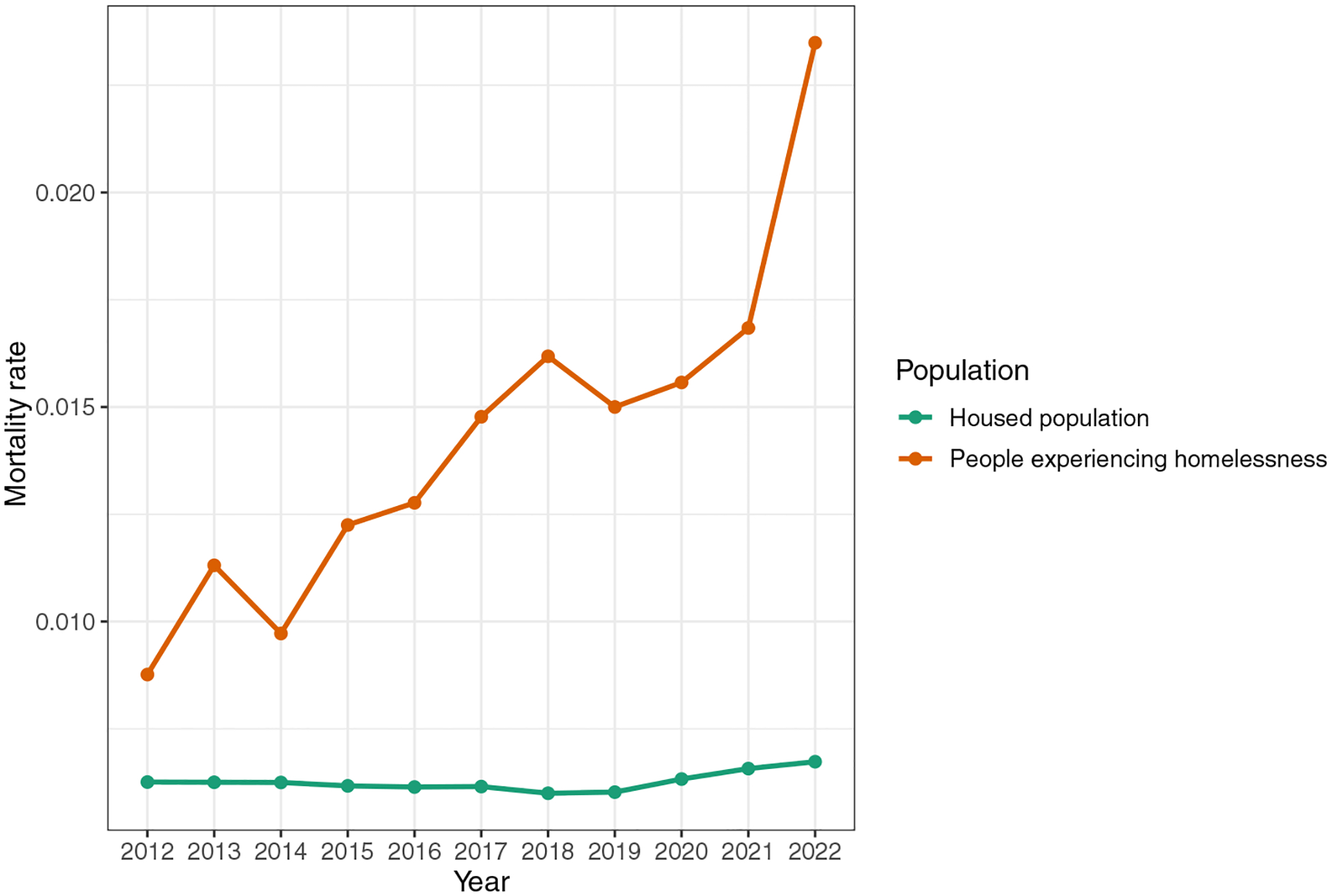
Unhoused vs housed death rates in King County, Washington Source: Created by the authors using the CDR for the unhoused population based on the yearly mortality information published by King County Public Health as the numerator (i.e., count of people experiencing homelessness who died on a given year), and the point-in-time count of people experiencing homelessness as the population denominator. The housed CRD was calculated using the yearly mortality statistics for King County and the American Community Survey estimate of yearly population. Notes: Crude death rate for King County, Washington comparing the housed population to the unhoused population.

**Figure 7:**
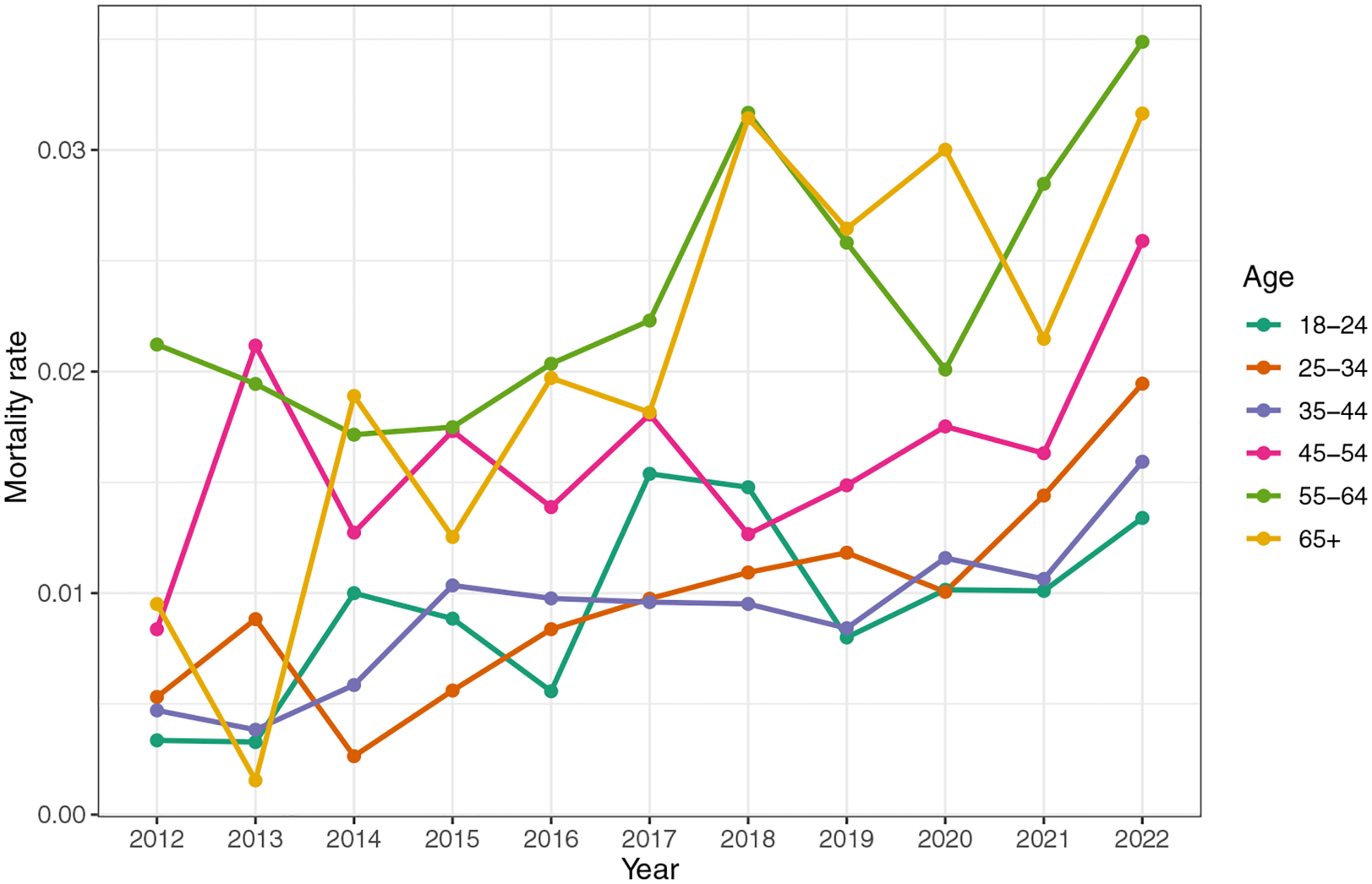
Age-adjusted death rates for people experiencing homelessness, King County, Washington Source: Created by the authors using the age-adjusted yearly mortality count for the unhoused population provided by King County Public Health as the numerator and the age-adjusted pointin-time count of people experiencing homelessness as the population denominator. The age structure for the point-in-time data was estimated from the age distribution in the 2023 unhoused population-representative survey of King County (Almquist et al., 2024b) because the point-intime data have only three age categories (under 18, 18–24, and over 24). Notes: Age-adjusted death rate for people experiencing homelessness in King County, Washington, comparing the housed population to the unhoused population.

In general, this is a complex problem with limited data. For example, King County Public Health (2025) provides death counts for people experiencing homelessness in King County, Washington, as labelled by the King County Medical Examiner. These data are among the best in the country, but are limited to the information available to the Medical Examiner (e.g., if the person was found in a tent, they will be labelled as homeless, but if they die in the ER, they may or may not be labelled as homeless). In Figure 6 we have computed the crude death rate (CDR) for people experiencing homelessness in King County, Washington, and the CDR for the housed population in King County. We can see that in 2022, the unhoused population had a mortality rate that was 3.49 times higher than that of the housed population. Obtaining age data for the unhoused population is complex, as the US Department of Housing and Urban Development only mandates age categories of “under 18”, “18–24” and “over 24”, and age data are not available for all years in which the point-in-time data are collected (available for 2007 to 2024, see US Department of Housing and Urban Development (2024)). In 2023, Almquist et al. (2024b) collected a population-representative survey of the unhoused population for King County, Washington, which can provide estimates of the age distribution for King County. If we applied those age distribution estimates across the 2012 to 2022 point-in-time data and combined them with age-recorded mortality data for people experiencing homelessness in King County, we could estimate the age- adjusted crude death rates (see Figure 7). Computing the resulting mean age at death from this process would result in a mean age of 50.5. Similarly, Scott et al. (2023) found that the average age of death for the unhoused population in King County was 50, compared to 80 for the housed population.

## Discussion

Even though the UN has recognised homelessness as a violation of human rights, and many organisations, countries, and other entities have described homelessness as a crisis (and some have even declared homelessness emergencies), there is no widespread agreement on homelessness definitions, best measurement practices or solutions (National Alliance to End Homelessness, 2024). In Europe and other parts of the world, homelessness continues to be a similarly complex issue, although the more robust welfare policies in Europe have contained the problem to some extent. If climate change damages urban housing infrastructure and leads to population displacement more generally, we can expect the ranks of the unhoused to increase. However, if we cannot measure homelessness accurately, we cannot monitor it or understand how social and classic demographers will incorporate it into census work. Furthermore, we are limited in our ability to obtain the high- quality metrics that are essential for demographic-style analysis, e.g., the mortality rate or the person-year effect on an individual of experiencing a week, a month, a year or 10 years of homelessness. There is ample evidence that many core demographic predictors are significantly influenced by spending a period of time without a home. Demographers are called to engage with the needs of people experiencing homelessness within all the classic areas of demography, including enumeration and forecasting, migration and forced displacement, life tables (entry/exit, spell and duration of time spent in homelessness) and mortality.

## Summary and future directions

The definitions, measurements and methods used for understanding the scope and composition of homelessness differ widely from region to region within a country, let alone across the world. This lack of general guidance, best practices and agreement on definitions makes it difficult to compare homelessness within or between countries, or to measure progress. This limits our ability to understand the scale of the problem (size) and the inequality of the problem (composition), and to isolate interventions that work (solutions to housing precarity and homelessness). Developing improved definitions, measurements and models for estimating the total numbers and demographics of people experiencing homelessness is, we believe, a central function of *core demography*, and we argue that the field should work to fill this large hole in the literature. We point to the need for standardisation in time scales (e.g., the number of people experiencing homelessness on a given night versus a year); in how we count (and in who we count); in how we define entry into and exit from homelessness (i.e., life tables of experiencing homelessness); and in how homelessness affects core demographic measures such as mortality (e.g., standardisation in the measurement of deaths attributed to homeless individuals and/or in population totals and the age structure of people experiencing homelessness is needed for mortality rates and age-adjusted rates), fertility (e.g., births while homeless) and migration (e.g., voluntary, international and forced by local authorities). New methods, such as those built on network science methods, represent exciting steps forward in finding new ways to count the unsheltered population; we direct researchers to look at Almquist et al. (2024b), which has become the official method for counting the unsheltered population in King County, Washington, and other studies on the use of network-based methods for understanding the unhoused communities in the US (Almquist, 2020; Almquist et al., 2024a; Anderson et al., 2021, 2024).

Finally, we end on the work of Colburn and Aldern (2022), who have demonstrated, in the context of the US, that homelessness is primarily driven by a lack of affordable housing, and have suggested that the best path forward is to increase housing units across the economic spectrum. Issues around affordable housing are likely to be exacerbated by major climate events; for example, in California, the Internal Displacement Monitoring Center estimates that in 2019, more than 300,000 people fled their homes in the face of wildfires (Internal Displacement Monitoring Centre (IDMC), 2019), and many were permanently displaced. This confluence of constrained housing supply and the destruction of housing attributable to climate change (e.g., wildfires in the US; McConnell et al., 2024) will likely exacerbate the homelessness problem and increase the need to understand how the lack of housing in the US and globally drives health inequities. In the contexts of both housing and homelessness, demographers can provide standardisation around measurement, definitions and future directions to facilitate the development of effective policy interventions.

## Figures and Tables

**Table 1:** Housing and Urban Development definition of homelessness

Category	Definition
Literal homelessness	Individuals and families who lack a fixed, regular, and adequate nighttime residence, including: Those staying in emergency shelters Those staying in transitional housing Those sleeping in places not meant for human habitation (e.g., streets, cars, parks); and Those exiting an institution where they resided for up to 90 days and were homeless before entering the institution
Imminent risk of homelessness	Individuals and families who will imminently lose their primary nighttime residence provided that: The residence will be lost within 14 days of the date of application for homeless assistance No subsequent residence has been identified; and The individual or family lacks the resources or support networks needed to obtain other permanent housing
Homeless under other federal statutes	Individuals and families who: Are fleeing, or attempting to flee, domestic violence Have no other residence; or Lack of resources or support networks to obtain other permanent housing

Source: Housing and Urban Development (2023).

Notes: The US federal government, Housing and Urban Development agency definition of homelessness.

**Table 2:** Estimates of doubled-up population King County, Washington, in 2018 and 2019

Year	Estimate	Total
2018	American Community Survey	15,984
	National Center for Education Statistics	10,004
	King County Public Health	5,009
2019	American Community Survey	11,711
	National Center for Education Statistics	13,496
	King County Public Health	4,214

Source: Created by the authors based on data from the American Community Survey and Integrated Public Use Microdata Series (Ruggles et al., 2024), the National Center for Education Statistics and King County Public Health. Doubled-up totals were estimated for King County, Washington, in 2018 and 2019 by summing up the King County Public Health database with deduplication; the National Center for Education Statistics student count with a census multiplier ((average family size with kids)/(average number of kids per family) = 3.01/1.79 = 1.68); and Richard et al. (2024) American Community Survey method.

Notes: Comparison of the doubled-up population in King County, Washington, for 2018 and 2019 estimated from different source information.

**Table 3:** Comparison of alternative methods for counting “street” homelessness

Method	Description
**Databases, passive data collection and the service community**	
Homeless Management Information System databases	Centralised databases at the continuum of care level cover most service activities. They do not collect data specifically on those living unsheltered or not using services.
Census data linkage	The American Community Survey and the decennial census collect information on people experiencing homelessness and can be linked to the Homeless Management Information System. Coverage of the population of people using shelters is high (around 90%), but people experiencing homelessness who live outside the shelter system are not covered.
Big data/cell phones	Cell phone and app data, such as those collected by Safegraph or other data aggregators, are used.
Survey of service workers	Service workers are surveyed after the unsheltered point-intime count to improve the final count.
**Survey and administrative data**	
Plant-capture/recapture methods	Evenly dispersed human decoys are used to estimate how many people are missed in the visual unsheltered point-intime count.
Capture-recapture (multiple-list) methods	A statistical approach is used to estimate the total population size by examining the differences between two or more lists.
Post unsheltered point-in-time count survey	A survey is conducted after an unsheltered point-in-time count to estimate demographic percentages. This method can be used to estimate the number of people who were missed.
**Visual unsheltered point-in-time technology improvements**	
App Geographic Information System tools	A geographic app with real-time GPS capabilities is used to improve the visual unsheltered point-in-time count.
**Online surveys and network methods**	
Network scale-up methods	Standard network scale-up methods use landline, cell phone, or address-based random population samples to pose network questions about how many unsheltered people the respondent knows. The responses are then scaled up to a population estimate for the unsheltered population.
Online surveys through marketing platforms	Apps such as Facebook are used to survey either the general population or the target population, and are combined with network scale-up methods to generate estimates. This approach is similar to the method proposed in Feehan and Cobb (2019).
**Social network methods**	
Respondent-driven sampling	Network referral methods are employed to produce a quasiprobability sample by tracing through the network of people experiencing homelessness in the area under study. This approach was used in the official King County, Washington, point-in-time count in 2022 and 2024.

Source: Created by the authors.

Notes: Table listing the methods proposed and used to estimate the number of unsheltered people experiencing homelessness.

## Footnotes

1 The US Department of Agriculture has had a long-standing interest in issues around feeding people experiencing homelessness in the rural areas in the US (Burt and Cohen, 1988).

2 King County, Washington, is the 12th largest US county, with a population of around 2.2 million people and the city of Seattle is located within its borders. It includes urban, suburban, and rural areas, as well as national wilderness.

3 We discuss this more in the following sections, but the basics are that continuum of care areas, directed by Housing and Urban Development’s agency, have developed a standard framework known as the point-in-time count to obtain a count of people living outside. The point-in-time is a collection of methods for estimating the number of people living unsheltered on a single night in January (as mandated by the Housing and Urban Development agency). Typically, this takes the form of a visual census administered by the continuum of care with the help of volunteers over several hours in the middle of the night.

4 Estimates of the total number of people living doubled-up in King County are derived from National Center for Education Statistics (2025) with a multiplier from the American Community Survey of (average family size with kids)/(average number of kids per family) = 3.01/1.79 = 1.68.

5 Bergmann et al. (2021) also has an excellent summary of alternative point-in-time strategies in the context of the continuum of care of Hennepin County, Minnesota.

6 Approved by the University of Washington IRB in 2022 (Study ID: STUDY00015310).

7 O’Donnell (2020) attempted to estimate the Australian national prevalence of people experiencing homelessness from the General Social Survey for Australia; we are unaware of a similar attempt to do so for the US in the US General Social Survey.
